# The Construction and Application of a Digital Coal Seam for Shearer Autonomous Navigation Cutting

**DOI:** 10.3390/s24175766

**Published:** 2024-09-05

**Authors:** Xuedi Hao, Jiajin Zhang, Rusen Wen, Chuan Gao, Xianlei Xu, Shirong Ge, Yiming Zhang, Shuyang Wang

**Affiliations:** 1 School of Mechanical and Electronical Engineering, China University of Mining and Technology (Beijing), Beijing 100083, China; 2 Key Laboratory of Coal Mine Intelligence and Robot Innovation Application Emergency Management Department, China University of Mining and Technology (Beijing), Beijing 100083, China; 3 School of Geoscience and Surveying Engineering, China University of Mining and Technology (Beijing), Beijing 100083, China

**Keywords:** intelligence, fully mechanized coal mining face, coal seam digital model, geological model, dynamic update mechanism

## Abstract

Accurately obtaining the geological characteristic digital model of a coal seam and surrounding rock in front of a fully mechanized mining face is one of the key technologies for automatic and continuous coal mining operation to realize an intelligent unmanned working face. The research on how to establish accurate and reliable coal seam digital models is a hot topic and technical bottleneck in the field of intelligent coal mining. This paper puts forward a construction method and dynamic update mechanism for a digital model of coal seam autonomous cutting by a coal mining machine, and verifies its effectiveness in experiments. Based on the interpolation model of drilling data, a fine coal seam digital model was established according to the results of geological statistical inversion, which overcomes the shortcomings of an insufficient lateral resolution of lithology and physical properties in a traditional geological model and can accurately depict the distribution trend of coal seams. By utilizing the numerical derivation of surrounding rock mining and geological SLAM advanced exploration, the coal seam digital model was modified to achieve a dynamic updating and optimization of the model, providing an accurate geological information guarantee for intelligent unmanned coal mining. Based on the model, it is possible to obtain the boundary and inclination information of the coal seam profile, and provide strategies for adjusting the height of the coal mining machine drum at the current position, achieving precise control of the automatic height adjustment of the coal mining machine.

## 1. Introduction

Intelligent, fully mechanized mining technology is a research hotspot in the coal industry. At present, China has gradually overcome a series of technical challenges of the remote control visualization of mining equipment in a fully mechanized coal mining face, a so called intelligent mining mode by automatic control and remote miner intervention [1]. However, coal and rock identification technology has not matured yet. Fully mechanized mining equipment can realize intelligent and continuous operation under simple coal seam geological conditions, but the coal seam geological information cannot be accurately sensed, and a shearer cannot realize coal seam self-adaptation cutting in complex geological conditions. Automated shearer cutting drum horizon control is a technical obstacle that limits the application of intelligent mining [2]. Ge et al. proposed the principle of shearer autonomous navigation and cutting, which is called the fourth technical stage of the shearer cutting methods [3]. The autonomous navigation and cutting indicate the building of an accurate coal seam digital model as the planned cutting paths of the shearer drums. The shearer can perform an unmanned coal cutting according to the planned paths in real time. Therefore, the completeness of the geological information of the working face coal seam is critical for the realization of the shearer autonomous navigation and cutting.

Currently, an integrated construction method of a digital geological model of a working face coal seam has been developed by using 3D seismic exploration, surface and downhole surveying, and some other methods in pre-mining and mining processes [4,5]. Among them, the high-resolution 3D seismic exploration technology established by Peng has broad applications for small coalfield structures [6,7,8]. Three-dimensional seismic exploration data have the characteristics of wide coverage, large detection depth, and high lateral resolution [9,10,11]. In traditional geological modeling methods, which rely on borehole data, the small size and uneven distribution of borehole data often result in low modeling accuracy, while it can provide abundant information with high vertical resolution. The key to constructing high-precision geological models lies in how to obtain high-resolution geological information and integrate various types of geological and engineering information [12].

The digital working face coal seam geological models constructed are traditionally mostly static. However, in the process of coal cutting, the geological and environmental information of the working face continuously changes, and the dynamic update of the 3D working face geological model is inevitable [13,14,15,16]. Sun proposed the key technology of a dynamic correction of the 3D geological model of a coal mine [17]. Yin proposed an accuracy evaluation method and updating technology for the 3D geological model based on the entropy weight method, and utilized the weights of influencing factors to perform local updates and the reconstruction of changing data [18]. But the practicality of the dynamic correction of these 3D geological models for the complex conditions mentioned above needs more research.

This paper proposes a building method of a digital coal seam model with self-adaptive regulation and a dynamic update for a geological environment in a fully mechanized mining working face. The geostatistical inversion of borehole and geological data is used to establish high-resolution 3D volume data with equal attribute probability, which are then subjected to a difference analysis and preliminary optimization for an accurate correction of the coal seam. To address the issue of an inability to interact with dynamic underground mining data, a numerical calculation of surrounding rock and random correction of the geological SLAM technique are employed for real-time updating of the model. This method provides optimized cutting trajectories for the shearer cutting, as it ultimately lays the foundation for coal seam geological adaptive and intelligent mining operations. And the real-time data provided by the coal seam navigation model provide adjustment strategies for intelligent height adjustment of the shearer drum in the current position state.

## 2. Construction of Refined Coal Seam Digital Model

### 2.1. Geostatistical Inversion to Predict Coal Thickness

The refined coal seam digital model is created by combining the geostatistical inversion results with the vertical high-resolution borehole information. This approach fully exploits the lithological information contained within the seismic data to optimize the vertical resolution of the 3D seismic inversion data. Conventional seismic interpretation and deterministic inversion results may fail to distinguish thin coal seams, but the geostatistical inversion method can accurately invert a thin coal seam with a thickness of 1 m in the model.

The geostatistical inversion method based on the Markov chain and Monte Carlo (MCMC) algorithm integrates geological, well logging, seismic, and other information [19]. It employs Bayesian discrimination, Markov chain sampling, and Monte Carlo simulation techniques to achieve this integration. The theory meets the high-resolution lithological inversion standard required for coal mine production and provides a basic framework for establishing “transparent mines”. The MCMC algorithm’s basic principle is to use statistical sampling to make the Markov chain converge apply to the high-probability-density area by establishing a stable distribution P(x) and continuously updating the sample information. In essence, the MCMC algorithm uses the Monte Carlo integral of the Markov chain, where the Markov chain samples from the target distribution and Monte Carlo represents the application of the Monte Carlo integral method based on the sample drawn. The estimated value of the requested parameter can be determined. Modeling based on geostatistical inversion results is achieved through combining the advantages of 3D seismic data and borehole data using the geostatistical inversion method [20]. This approach has the features of a high horizontal resolution of 3D seismic data and high vertical resolution of borehole data. Finally, with high-resolution lithological data, establishing a high-resolution coal seam model is successfully realized.

**Figure 1:**
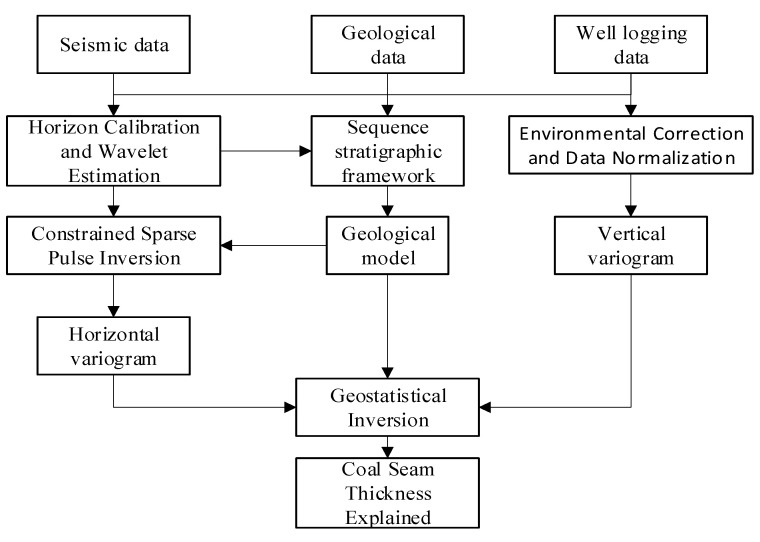
Coal thickness prediction process based on geostatistical inversion.

The following three steps are required to complete the geostatistical inversion. Firstly, process the logging information, geological data, and seismic data. Secondly, use statistical inversion to refine the lithology information. Thirdly, obtain predicted coal thickness. The process of the geostatistical inversion and prediction of coal thickness is shown in Figure 1. Logging data are easily affected by objective conditions and require data preprocessing. The corrected data are synthesized into the wave impedance curve and a statistical analysis is performed on it to convert the data. To generate the wave impedance data conforming to the Gaussian distribution, an initial model is generated and then a variogram is established. Compared with other inversion methods, logging data and seismic wave impedance data can be used separately to establish horizontal and vertical variograms, which can provide geological space at the same time. The fitting accuracy of each azimuth is determined. And finally, control parameters are determined. The optimal state results of the synthetic data are achieved through iteration to realize the interpretation of the coal seam thickness.

**Figure 2:**
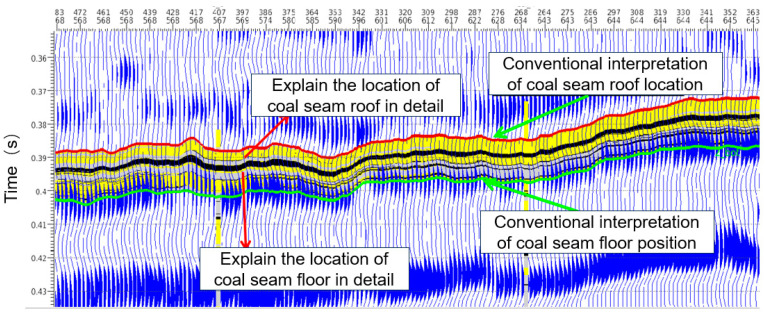
Detailed explanation of the position of the roof and floor of the coal seam.

As shown in Figure 2, the refined coal seam roof and floor interpretation exhibits a resolution consistent with the actual coal seam distribution trend. Moreover, coal seams significantly lower than the theoretical resolution of conventional interpretation can be accurately identified. Compared to the base plate position, accuracy has been significantly improved. Once the fine interpretation of the coal seam roof and floor is achieved, the thickness of the coal seam can be predicted. Additionally, changes in the thickness of the coal seam and the ratio of various lithologies are correlated with changes in seismic data and P wave impedance data. The correlation between changes in the thickness of the coal seam and the ratio of various lithologies is mainly realized through the following steps: identifying the lithological boundary of the roof and floor, tracking the event axis, calculating the distance between the roof and floor, and obtaining the coal seam thickness from the 3D velocity volume model, which is obtained from the deterministic inversion results. In conclusion, it is reasonable to use geostatistical inversion for coal seam thickness prediction.

### 2.2. Example of Creating Refined Coal Seam Digital Model

Before establishing a refined coal seam digital model based on the geostatistical inversion, the mining engineering software 3D Mine (Version Number: 2016) was used to establish an initial model with borehole interpolation. The software 3D Mine employs the method of triangular network modeling to build a drilling database with scattered drilling information and combines fault lines and profiles to create an initial model. However, the initial coal seam model established by 3D Mine does not achieve sufficient accuracy, and it is necessary to use the 3D velocity model established by seismic inversion to accurately correct the initial model. The exact match between drilling and 3D seismic depth can be solved by using a fine well-seismic calibration of the 3D data set. The time-to-depth conversion of the spatial 3D data set is the key to directly building a coal seam model using the seismic inversion result data. Compared with conventional deterministic inversion, pre-stack geostatistical inversion results have a higher vertical resolution. The key is to have the same sampling rate for the logging data, and the inversion can be directly used to obtain higher resolution. Shear wave and P wave layer velocity sets are also obtained through this process.

**Figure 3:**
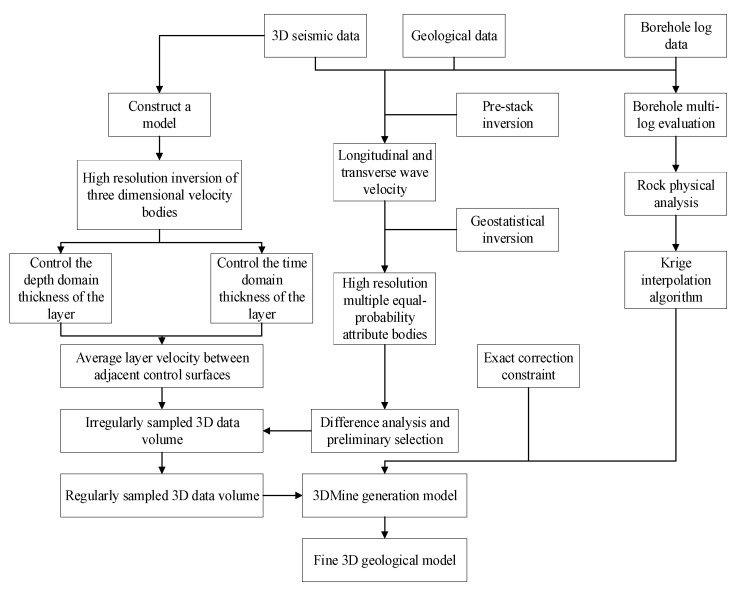
Flow chart for constructing refined coal seam digital model.

Geostatistical inversion is performed with seismic data, borehole data, and geological data to establish the high resolution and equal probability data set of various attributes, which is the basis for different analyses and preliminary optimization of 3D coal seam data. The data set is used for a real-time depth conversion of the 3D seam model. The initial coal seam model established by 3D Mine is not particularly accurate in the thickness of the area where the boreholes are located, and the lateral resolution is low. The 3D velocity model is automatically divided by the depth domain grid to accurately calibrate the depth of the thin layers between the controlled layers. Based on a high lateral resolution of the 3D seismic interpretation itself, a 3D velocity model with a more accurate velocity structure between strata layers is finally obtained. At the same time, precise correspondence between the drilling depth and the velocity control layer in the depth domain is ensured. The construction flow chart of the fine coal seam model is shown in Figure 3.

#### 2.2.1. Mining Area Geological Overview

**Figure 4:**
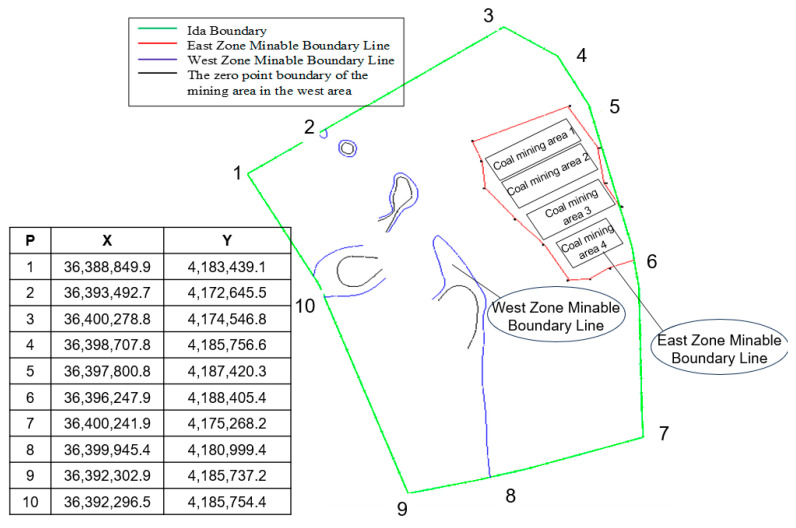
Geographical location and mining situation of coal mine.

Taking a certain mine as an example to verify the method, the geological conditions of this mine are very complicated. The average thickness of the coal series is 283.03 m, with 15 layers of numbered coal seams. The total coal thickness is 29.26 m, of which 10 layers can be mined. In the example, the modeling is for coal seam 4-1, 4-2, and 4-3. Coal seam 4-1 is positioned at the top of this segment, averaging 4.99 m in thickness. Coal seam 4-2 is situated in the middle of this segment, averaging 3.48 m in thickness, and its upper surface averages 11.62 m from the lower surface of coal seam 4-1. Coal seam 4-3 is located at the bottom of this segment, with its upper surface averaging 21.84 m from the lower surface of coal seam 4-2, and averaging 4.34 m in thickness. The geographical location and mining conditions of the coal mine are shown in Figure 4.

#### 2.2.2. Initial Seam Model Construction

**Figure 5:**
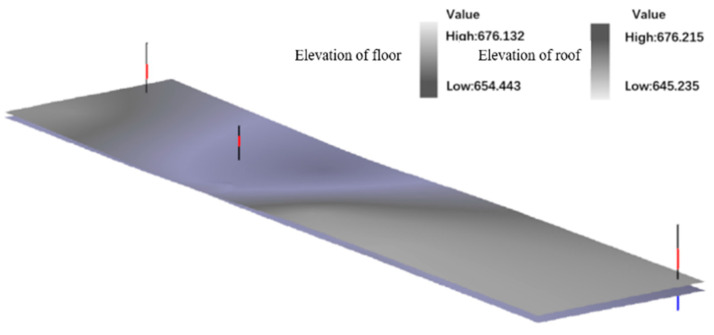
Schematic diagram of initial coal seam model of 110,301 working faces.

The mining engineering software 3D Mine is used to establish the initial model with borehole interpolation. By establishing the borehole database of a certain mine, combined with the two-dimensional geological model, the determination of the coal seam boundary of the working face is completed. Modeling of the entire coal seam and extracting lithological information are used to establish a coal seam model. Figure 5 shows the three-dimensional display of the boreholes and the coal seam 4-1 in three mining areas inside the experimental working face of a mining area.

#### 2.2.3. Roof and Floor Fine Interpretation of Coal Seam Thickness Prediction

**Figure 6:**
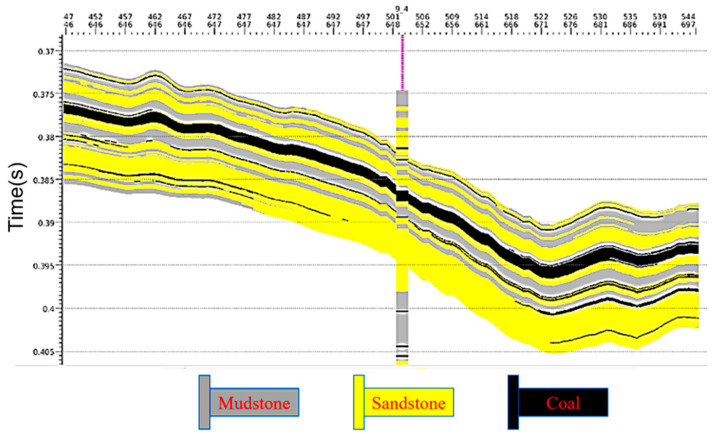
The 4-1 coal seam lithology inversion results.

**Figure 7:**
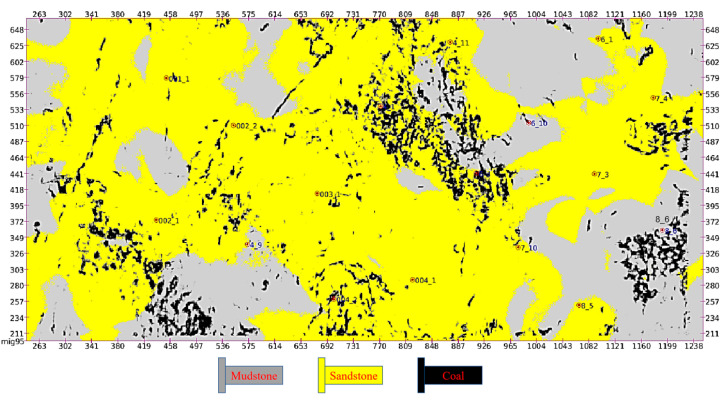
The 4-1 overlapping distribution model of the coal roof and floor lithology and thickness.

Figure 6 shows the lithology inversion results of coal seam 4-1. The roof and floor stratum of the coal seam are mainly composed of mudstone and sandstone, which accurately reflect the positions of the roof and floor of the coal seam and its tendency. Figure 7 shows the superimposed distribution of lithology, and thickness of the roof and floor in seam 4-1. The geostatistical inversion results integrate the lithology, physical properties, and thickness characteristics of the coal seam to achieve an accurate acquisition of high-resolution lithologic data.

Taking the mining starting point as the origin of the coordinate system, the direction of the working face is along the X-direction and the advancing direction of the working face is along the Y-direction. The inversion coal thickness prediction information of the experimental working face is obtained from the results of the fine interpretation of the roof and floor of the coal seam in Table 1.

#### 2.2.4. Initial Seam Refinement

**Figure 8:**
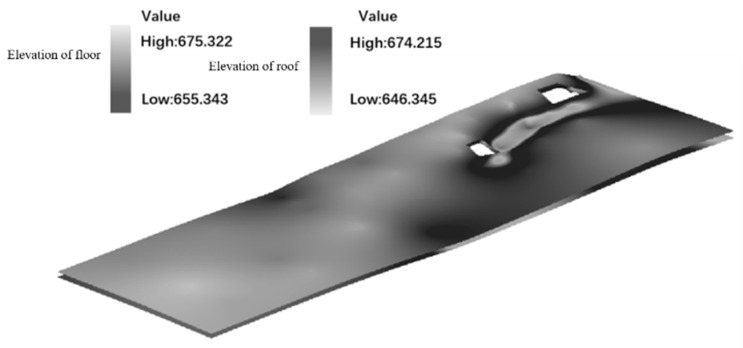
Schematic diagram of fine coal seam model of 110,301 working faces.

Based on the initial coal seam model, the refined construction of the experimental working face profile was realized. On the 3D Mine platform, using the floor DEM of the initial coal seam model and combined with the coal seam thickness prediction results obtained as mentioned earlier and the plane coordinates of the sampling points, the elevation information of the geophysical points was obtained. The Kriging interpolation algorithm was then used to establish the DEM of the coal seam roof. As shown in Figure 8, the model uses grid cells to store data information, with a size of 0.8 m × 0.8 m for each cell. During the modeling process, the number of grid lattices of the coal seam in the statistical working face was 2512 and the number of columns was 312. The elevation ranges of the roof and floor of the coal seam are 655.343–675.322 m and 646.345–674.215 m, respectively. The established model is consistent with the actual fitting degree, with a difference in interpretation thickness of 0.05 m from the roof and to the floor of the coal seam.

#### 2.2.5. Accuracy Comparison

**Figure 9:**
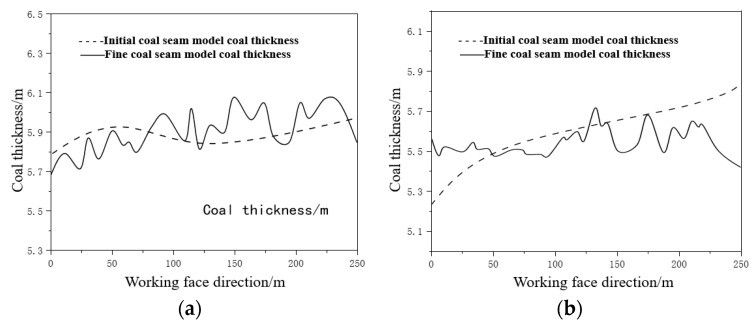
Prediction and comparison curve of coal thickness of 5 m in working face (**a**); prediction and comparison curve of coal thickness of 105 m in working face (**b**).

The data points of the refined coal seam model advancing 5 m and 105 m along the direction of the working face are presented in Table 2 and Table 3. The coal thickness curves of different advancing distances in the working face are shown from the initial coal seam model and the fine model in Figure 9. It shows that the numerical smoothing calculation of coal thickness established by borehole data can only provide a general understanding of the coal seam trend, while the value of coal thickness predicted by geostatistical inversion fluctuates within a small range. This is because the number of boreholes is limited and the distribution is scattered, resulting in high vertical resolution but low lateral resolution, which fails to reflect the actual change in coal thickness accurately. On the other hand, high-longitudinal-resolution information can accurately invert coal thickness information, providing a more detailed description of the coal seam trend, which meets the need of fine coal seam construction.

## 3. Seam Model Update

With the progress of underground mining, strata geological data are constantly changing. The original statistical seam model lacks a mechanism of interacting with the dynamic data of underground mining. It is necessary to explore methods for efficient and timely updating of the coal seam model. The update of the seam model is based on numerical DEM calculation results of the surrounding rock, combined with real-time geological detection by SLAM to obtain geophysical information ahead of the working face. By comprehensively correcting and updating the coal seam model in real time by integrating stress field, deformation field, and lithological characteristics of the roof and floor of the coal seam, the seam model can be updated in a timely manner.

### 3.1. Model Update Based on Numerical Calculation Results of Surrounding Rock

**Figure 10:**
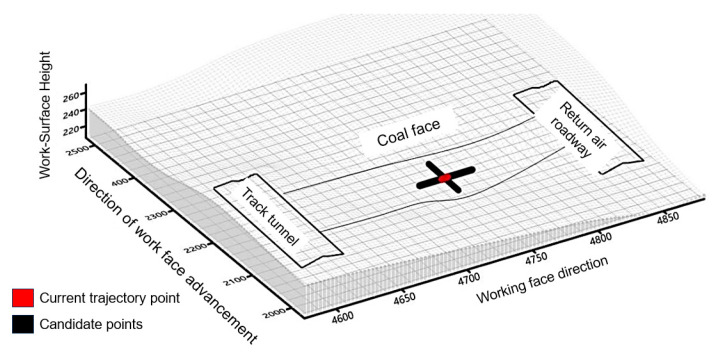
Schematic diagram of coal seam grid index.

**Figure 11:**
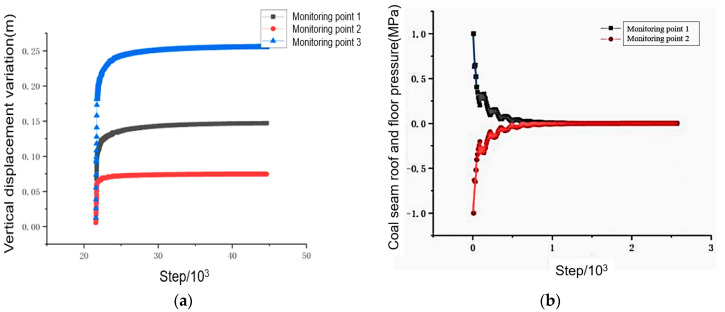
The simulated pressure variation curve of the surrounding rock at a mine (**a**); the simulated deformation variation in the monitoring points of the surrounding rock at a mine (**b**).

As shown in Figure 10, the working face to be mined is depicted. The numerical simulation object for this time is the entire working face of a length of 270 m. The step interval is set at 10 m/step, with a total of 40 steps to advance. Additionally, three monitoring points are positioned on the top and bottom of the coal seam along the direction of the working face. Figure 11a shows the vertical displacement curves of monitoring point 1, 2, and 3. During the initial stage of coal seam mining, both the roof and floor coal seams show small vertical displacement values. However, as the mining process continues, the impact of mining on the displacement of the surrounding rock increases, leading to a continuous displacement increase. When the working face advances a certain distance, the vertical displacement of the top and bottom strata stabilizes. Figure 11b displays the vertical stress curve for monitoring point 1 on the roof and monitoring point 2 on the floor. The roof and floor stress gradually decrease with the mining process, which is due to the increased stress area during coal seam mining. The mined area then becomes the stress relief area, while the rock formation forms the bottom plate of the hollow part.

**Figure 12:**
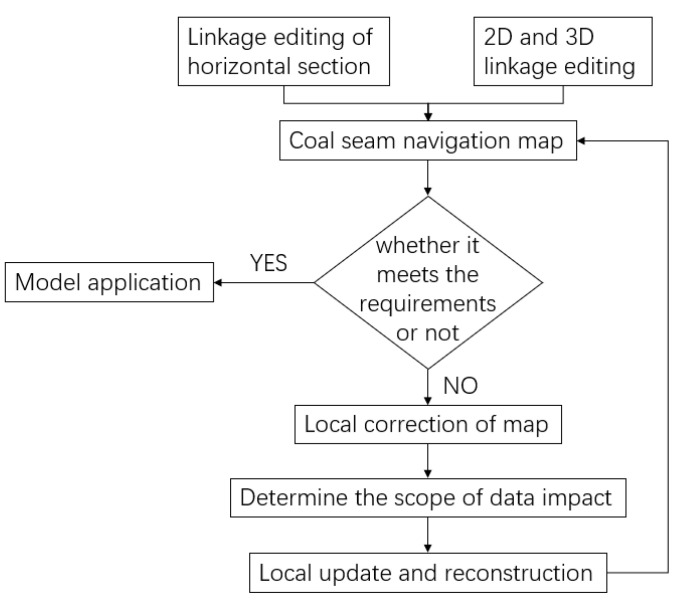
Schematic diagram of coal mining on multiple seam models.

The coal seam model is imported into the numerical analysis software FLAC3D (Version Number: 5.0) through the interface program. The numerical results of the surrounding rock under normal in situ stress on the roof and floor, as well as the vertical stress and deformation after mining according to the cutting path of the shearer, are obtained through numerical calculation. The model updating process based on the numerical calculation results of surrounding rock mining is shown in Figure 12. In this process, it is necessary to use flat section linkage editing technology, 2D linkage editing technology, and 3D linkage editing technology, all of which share the common feature of updating data at a certain point in the 2D plane and then updating the 3D model. The flat section linkage editing technology processes the section of the coal seam digital model, uses the numerical calculation results of surrounding rock mining to obtain the change data of the coal seam roof and floor lines, and then updates and corrects the section. The coal seam digital model corresponding to the 2D section will also be updated.

### 3.2. Simultaneous Positioning and Model Update in Underground Mining

To improve the accuracy of the model, a second update is performed using the C-SLAM (Coal-seam Simultaneous Localization and Mapping) technology. This technology can achieve detection depths of up to 10 m and recognition accuracy of typically 5 cm, providing navigation coordinates for the 3~5 cuts of the shearer. Specifically, based on the coal rock horizon intelligent detection system, the automatic identification and real-time tracking of the coal rock interface are achieved. The coal rock interface information is obtained in real time through dynamic monitoring, and the precise correction value of this data is then inserted into the fine coal seam cutting digital model to complete simultaneous positioning and model updates. This ensures that accurate data of the structural surface of the roof and floor of the coal seam are obtained.

The C-SLAM technology employs noncontact radar technology to detect the local structure of the coal seam in the working face. The detection antenna is kept at a distance of 300–400 mm from the coal wall of the working face, and continuous scanning and precise positioning along the coal wall enable the collection of the local coal seam structure and undulation data with a three-dimensional accuracy of up to 50 mm. The collected data are automatically processed in real time and wirelessly transmitted to the shearer, providing a precise digital model for the shearer cutting.

**Figure 13:**
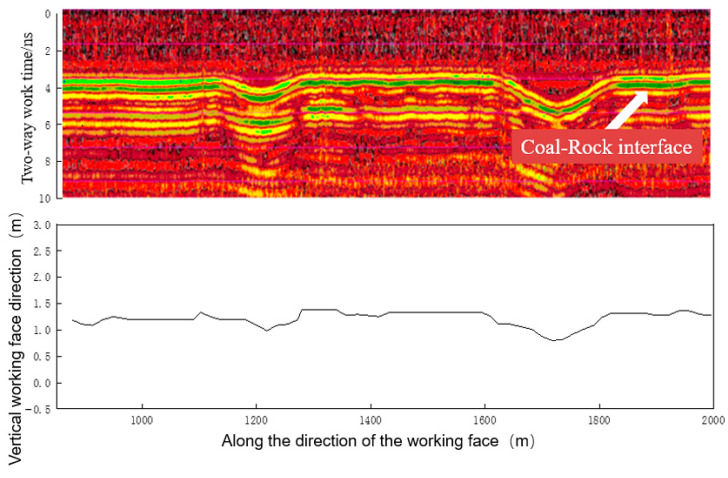
Shendong Jinjie Coal Mine exploration experiment.

The core component of the C-SLAM technology is Ground Penetrating Radar (GPR) technology, which involves transmitting high-frequency electromagnetic waves from the antenna along with the shearer through continuous moving. During propagation, the electromagnetic wave signal encounters electrical difference strata (such as differences in dielectric constant) and undergoes reflection, transmission, and refraction at the medium interface. The radar host accurately records the two-way travel time, amplitude, and phase of the reflected wave, resulting in the acquisition of cross-sectional scanning radar images of the coal rock medium. This process enables the identification of coal rock layers and the dynamic acquisition of high-precision information of coal rock interfaces, coal seam thicknesses, roof and floor boundaries, coal seam undulation, geological structures, and other parameters. Figure 13 presents the results of a coal seam detection experiment conducted in Shen Dong Jinjie Coal Mine, where the coal rock interface detection accuracy was recorded at 50 mm. Through data interaction with the shearer control system and centralized control centers, effective drum height adjustment strategies can be formulated to achieve successful shearer navigation cutting.

## 4. Method for Autonomous Height Adjustment of Coal Mining Machines Based on Coal Seam Digital Models

Based on coal seam digital models and coal mining machine positioning technology, it is possible to achieve autonomous height adjustment of the coal mining machine drum. The intelligent height adjustment of the shearer drum in the direction of the working face utilizes the coal seam digital model to provide real-time top and bottom plate height lines. In the case of multi-information guidance, combined with decision algorithms, the shearer drum is cut along the top and bottom plate lines of the coal seam under certain principles or constraints. The geological conditions of coal seams are complex. In the direction of advancing the working face, the coal mining machine relies on the information of the coal seam inclination angle in the direction of advancing the working face to change the elevation mining posture with the undulation of the coal seam, so that the elevation mining angle is consistent with the coal seam inclination information provided by the digital model. The elevation amount is obtained from the coal seam inclination angle, and the adjustment amount is calculated by combining the cutting width of the coal mining machine to achieve intelligent elevation adjustment of the coal mining machine in the direction of recovery.

### 4.1. Autonomous Height Adjustment of the Coal Mining Machine in the Direction of the Working Face

**Figure 14:**
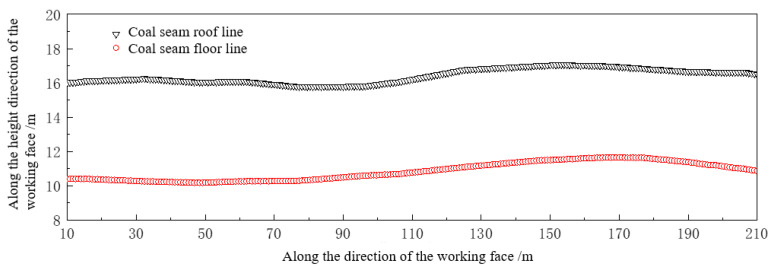
The working face advances the roof and floor line of the 200 m coal seam.

The shearer is assisted by the coal seam digital model to achieve autonomous height adjustment of the working face direction. In order to improve the recovery rate, while meeting the support requirements, the shearer follows the maximum cutting efficiency and needs to ensure that the drum cuts along the coal seam roof and floor as much as possible. At this time, accurate data on the height line of the roof and floor are required. The coal seam digital model established in the previous text provides the coal seam roof and floor line based on the current position of the coal mining machine, as shown in Figure 14, which is a 200 m coal seam roof and floor curve in the advancing direction. The coal mining machine can achieve autonomous elevation adjustment in the working face direction through navigation data.

### 4.2. Adjusting the Height of the Coal Mining Machine in the Direction of Advancing the Working Face

**Figure 15:**
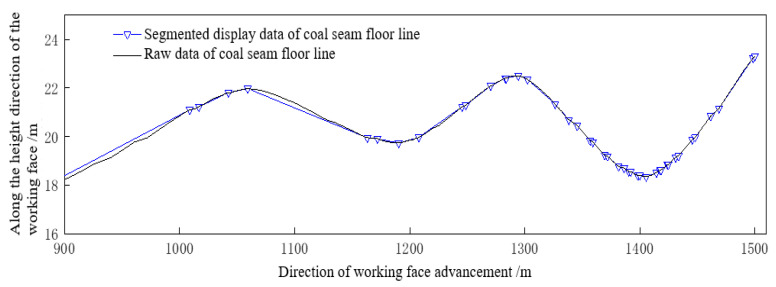
The original floor line and segment display of the coal seam (900–1500 m).

Based on the floor data obtained from the coal seam digital model, a segmented linear representation method for the coal seam floor can be established as shown in Figure 15, in order to obtain the slope of the segmented straight line of the coal seam floor, which is the coal seam dip angle.

**Figure 16:**
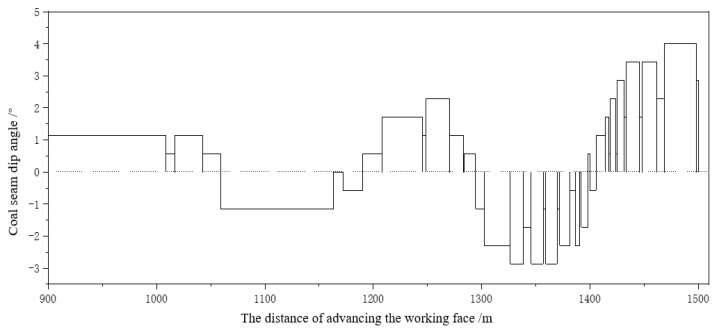
Δh = 0.01 m data display of inclination angle of coal seam floor.

The key to achieving adaptive height adjustment of the shearer drum is to control the attitude of the shearer to ensure that its pitch angle is consistent with the inclination angle of the coal seam. The amount of height adjustment of the coal mining machine in the direction of mining is related to two factors, namely the change in pitch angle and the cutting width. The control accuracy of the height adjustment of the coal mining machine is set to be δ, which is the minimum height adjustment value for the smooth operation of the coal mining machine. By using the angle parameters of the shearer rocker arm and the positioning and orientation system of the shearer, the current cutting trajectory of the shearer is calculated. Combined with the inclination angle of the coal seam, the adjustment amount of the next shearer’s bottom is obtained. When δ = 0.01 m, the inclination angle data of the coal seam in the direction of working face advancement from 900 m to 1500 m are obtained by the slope of the segmented straight line, as shown in Table 4 and Figure 16.

## 5. Conclusions

(1) The geostatistical inversion of lithological, seismic inversion and borehole data can result in a refined coal seam model. The identification resolution of a thin coal seam is about 1 m, which lays a reliable basis for cutting path design of a shearer.
(2) Using the numerical DEM calculation results of surrounding rock and the geological information in front of the working coal wall obtained by real-time detection by C-SLAM, the coal seam model can be corrected and updated in real time, resulting in a more precise coal seam profile limit. The detection depth can reach up to 10 m with an identification accuracy of approximately 5 cm. This refined model provides accurate navigation coordinates for three to five cuts of a shearer.
(3) Based on the navigation model, the boundary and dip angle information of the coal seam roof and floor is obtained, and the real-time positioning information of the coal mining machine is matched. Moreover, real-time data are provided through the coal seam navigation model to provide adjustment strategies for the intelligent height adjustment of the drum of the coal mining machine in the current position state, achieving precise control of the automatic height adjustment of the coal mining machine.

## Figures and Tables

**Table 1:** Inverse coal seam thickness prediction from experimental working face.

Number	The Direction of X/m	The Direction of Y/m	Range of Coal Seam Thickness/m
1	110–340 m	235–345 m	1.9 m to 2.7 m
2	230–500 m	0–230 m	2.5 m to 4.5 m
3	430–780 m	0–190 m	1.9–2.2 m
4	780–1300 m	0–238.56 m	2.4 m to 2.8 m
5	1300–1900 m	0–265.24 m	5.7 m to 6.1 m
6	1900–2400 m	0–265.24 m	5.3–5.7 m
7	2400–2780 m	0–265 m	4.9–5.5 m
8	2780–3160 m	0–265 m	4.9–5.5 m
9	3160–3450 m	0–265.24 m	5.3 m to 5.7 m

**Table 2:** Refined coal seam model data points for working face direction (advance 5 m).

The Serial Number	The Direction of Working Face/m	Roof Height/m	Floor Height/m	The Thickness of Coal Seam/m
1	−0.003	13.2752	7.56567	5.71375
2	0.797	13.2774	7.56274	5.71466
3	1.597	13.27545	7.55988	5.71557
4	2.397	13.2735	7.55701	5.71649
5	3.197	13.27148	7.55414	5.71734
6	3.997	13.26953	7.55121	5.71832
…	…	…	…	…
314	250.397	16.53276	10.66943	5.86333

**Table 3:** Refined coal seam model data points for working face direction (advance 105 m).

The Serial Number	The Direction of Working Face/m	Roof Height/m	Floor Height/m	The Thickness of Coal Seam/m
1	0.797	18.33044	12.76058	5.56986
2	1.597	18.34418	12.77584	5.56834
3	2.397	18.35797	12.79116	5.56681
4	3.197	18.37268	12.80746	5.56522
5	3.997	18.38739	12.82375	5.56364
6	4.797	18.40211	12.84005	5.56205
…	…	…	…	…
410	250.397	22.3974	16.94543	5.45197

**Table 4:** Δh  = 0.01 m dip angle data of coal seam floor (900–1500 m).

Number	Working Face Advance Distance/m	Coal Seam Dip Angle/°
1	1008.8–1016.2	0.57294
2	1016.2–1042.4	1.14576
3	1042.4–1059.2	0.57294
4	1059.2–1163.2	−1.14576
5	1172.0–1190.4	−0.57294
6	1190.4–1208.0	0.57294
7	1208.0–1245.6	1.71836
8	1245.6–1248.8	1.14576
9	1248.8–1270.4	2.29061
10	1270.4–1283.2	1.14576
11	1283.2–1284.0	0.57294
12	1284.0–1302.4	−1.14576
13	1302.4–1326.4	−2.29061
14	1326.4–1338.4	−2.86241
15	1338.4–1345.6	−1.71836
16	1345.6–1356.8	−2.86241
17	1356.8–1356.8	−1.14576
18	1356.8–1369.6	−2.86241
...	...	...
35	1498.4–1500	2.86241

## Data Availability

Data are contained within the article.
